# Asymmetric aza-Diels-Alder reaction of Danishefsky's diene with imines in a chiral reaction medium

**DOI:** 10.1186/1860-5397-2-18

**Published:** 2006-09-18

**Authors:** Bruce Pégot, Olivier Nguyen Van Buu, Didier Gori, Giang Vo-Thanh

**Affiliations:** 1 Laboratoire de Chimie des Procédés et Substances Naturelles, ICMMO, CNRS UMR 8182, Université Paris-Sud, 91405 Orsay Cedex, France

## Abstract

The asymmetric aza-Diels-Alder reaction of chiral imines with Danishefsky's diene in chiral ionic liquids provides the corresponding cycloadduct with moderate to high diastereoselectivity. The reaction has proved to perform better at room temperature in ionic liquids without either Lewis acid catalyst or organic solvent. Chiral ionic liquids are recycled while their efficiency is preserved.

## Background

**Scheme 1:**
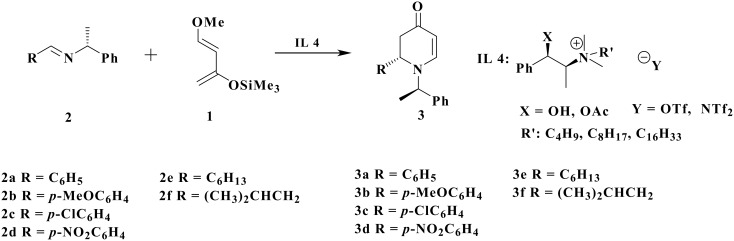
Asymmetric aza-Diels-Alder reaction of Danishefsky's diene **1** with imines **2**.

Aza-Diels-Alder reactions rank among the most efficient method for the construction of nitrogen-containing six-membered ring compounds.[1] The reaction of Danishefsky's diene **1** with imine **2** provides a convenient protocol for the synthesis of 2-substituted-2,3-dihydro-4-pyridones **3** (Scheme 1), which allow for important synthetic applications in natural and unnatural products alike.[2–3] Much progress has been made recently in these reactions and a number of Lewis acid-catalyzed versions in organic solvents have been reported. Thus, various Lewis acids such as BF_3_.Et_2_O,[4] ZnCl_2_,[5] or lanthanide triflates[6] and Brönsted acids, including HBF_4_ or TsOH[7] largely helped promote the reaction. Of late, catalytic asymmetric versions of the aza-Diels-Alder reaction have been explored and high stereoselectivities were reported. So far, a few catalyst systems have been quoted in relation to the asymmetric process. Among them are: the zirconium-binaphthol complexes developed by Kobayashi *et al*., [8–11] the silver catalysts of phosphine peptide Schiff bases reported by Snapper and Hoveyda,[12] as well as the copper complexes of BINAP and phosphino-oxazolidines described by Jørgensen,[13–14] and the chiral copper complexes of phosphino sulfenyl ferrocenes reported by Carretero.[15] The catalyst systems also include the Lewis acid catalysts based on boron-BINOL complexes [16–18] and lastly zinc-BINOL complexes developed by Whiting *et al*.[19] In all the above cases, chirality transfer was carried out using chiral Lewis acid catalysts. However, these are often expensive, toxic, and not easily recycled. Up until this point, no chiral solvent is reported to have been used in this transformation. Therefore, when researching chiral ionic liquids (ILs), we had two objectives in mind: to promote 'green reaction media' and most importantly to provide a highly efficient chirality transfer due to the high degree of organization of the chiral ionic liquids.

Chiral solvents are reported to have been already used as a sole inducer of enantiomeric excess in organic reactions. [20–22] However, the process resulted in low enantioselectivities not to mention the complication in preparing the solvent and the ridiculously high cost. It is, therefore, safe to say that chiral ILs are much more efficient than the traditional chiral solvent in asymmetric induction, due to the simplicity in their preparation, their recyclabilities and their unique properties.[23–24]

Thus, we were the first to promote the use of chiral ILs as the only source of chirality in the asymmetric Baylis-Hillman reaction.[25] Ee's of up to 44% were obtained using ephedrinium-based ILs.[26] Two further applications of chiral ILs in the asymmetric induction were reported in 2005 by Armstrong[27] and Bao.[28] Recently, Leitner and coworkers reported a highly enantioselective aza-Baylis-Hillman reaction (up to 84% ee) by using an IL with chiral anion, derived from (-)-malic acid, as the sole source of chirality.[29]

In connection with our studies on chiral ILs and our ongoing project on asymmetric synthesis, we describe herein how chiral ILs can be used as a new chiral reaction medium in the asymmetric aza-Diels-Alder reaction between Danishefsky's diene and chiral imines.

## Results

We have recently reported a highly efficient procedure for the synthesis of 2-substituted-2,3-dihydro-4-pyridone derivatives through the aza-Diels-Alder reaction under 'green chemistry' conditions. The reaction has been found to perform well at room temperature in ionic liquids using no Lewis acid catalyst or organic solvent.[30]

Encouraged by this result, we decided to develop asymmetric aza-Diels-Alder reactions of chiral imines with Danishefsky's diene using a chiral ionic liquid as a chiral reaction medium.

In our initial studies, we attempt to optimize the conditions for the aza-Diels-Alder reaction between Danishefsky's diene **1** with imines **2** (Scheme 1, R = Ph) in the presence of chiral IL **4** (X = OH, R' = C_8_H_17_, Y = OTf), easily obtained in 'two-step sequence' reaction from (*N*)-methylephedrine.[26] As reported by our laboratory,[31] the imine **2** was synthesized by condensation of benzaldehyde and (*R*)-(+)-methylbenzylamine under solvent-free microwave activation. All aza-Diels-Alder reactions were performed under argon at room temperature without either a Lewis acid catalyst or organic solvent (Table 1).

**Table 1:** Asymmetric aza-Diels-Alder reaction of Danishefsky's diene **1** with aromatic imine **2** (R = Ph) in the presence of chiral IL **4** (X = OH, R' = C_8_H_17_, Y = OTf) for 4.30 hours.

Entry	IL **4** (equiv.)	Diene **1**^a^ (equiv.)	Temperature (°C)	Conversion^b^ (%)	Yield **3**^b^ (%)	de **3**^c^ (%)

1	1	1.5^d^	30	72	48 (45)	51
2	1	1.5	30	67	53 (57)	52
3	0.5	1.5	30	53	44 (42)	43
**4**	**2**	**1.5**	**30**	**75**	**66 (65)**	**60**
5	4	1.5	30	72	68 (70)	58
6	1	1.5	0	76	66 (62)	50
7	1	1.5	50	66	50 (46)	40

a) Diene added to reaction medium in three phases: 0.5 equiv. at equal intervals. b) Conversion and yield estimated by GC using an internal standard (octadecyl acrylate), isolated yields are given in brackets. c) de determined by chiral HPLC with a margin of error about 1%. d) 1.5 equiv. of diene added into reaction medium in one portion.

As illustrated in Table 1, both yield and diastereoselectivity are highest when performing the experiment with 2 equivalents of IL **4** and 1.5 equivalents of Danishefsky's diene for 4.5 hours. The two diastereomers obtained were separated by column chromatography and the assignment of the absolute configuration of the major product **3** was determined by comparison of the optical rotation and NMR spectra data with the literature values.[17]

Because of its strong tendency to decompose during the course of the reaction, Danishefsky's diene is not added all at once but rather is added to the reaction medium in three phases, 0.5 equivalents at equal intervals. With a single addition of 1.5 equivalents of diene, only 45% of yield was observed (entry 1). On the other hand, the slight excess of chiral IL leads to a noticeable enhancement in diastereoselectivity (entry 3 and 4). Moreover, no significant effect was observed when using a large excess of chiral IL (entry 5).

We further examined the effect of temperature on the reaction using the stoichiometric IL **4** (X = OH, R' = C_8_H_17_, Y = OTf) for 4.5 hours. The de dropped from 53% (Table 1, entry 2, 30°C) to 39% (Table 1, entry 7, 50°C) with only a small concomitant reduction in yield. On the other hand, no effect on diastereoselectivity was observed at 0°C (Table 1, entry 6). However, the best yield was obtained due to a reduction of Danishefsky's diene degradation at that temperature.

Recently, Li and coworkers reported the aza-Diels-Alder reaction of 4-iodo-2-trimethylsilyloxy-butadiene with chiral imines.[32] Good yields and comparable de's were obtained using the (*S*)-(-)-methylbenzylamine chiral auxiliary. However, all reactions had to be performed in an organic solvent under an inert atmosphere and required stoichiometric loadings of Lewis acid promoter, the use of which is not recommended under 'green chemistry' conditions.

Having established the high efficiency of chiral ILs is a 'green method' for the chirality transfer in the aza-Diels-Alder reaction, we proceeded to elaborate the IL structure effect. To that end, a series of different alkyl chain lengths were tested on this model reaction (Table 2). Surprisingly, contrary to results obtained in the asymmetric Baylis-Hillman reaction previously reported,[25] the de dropped from 60% (entry 2, R = C_8_H_17_) to 45% (entry 1, R = C_4_H_9_) and increased to 72% (entry 3, R = C_16_H_33_) when performed in similar conditions. However, a very low yield was obtained in the case of R = C_8_H_17_, which may be explained by a heterogeneous reaction medium whereby the salt is solid at room temperature. On the other hand, no effect on diastereoselectivity was detected when using different anions (Y = OTf, NTf_2_). Because of the Danishefsky's diene decomposition in the presence of F^-^ anions (from BF_4_^-^ and PF_6_^-^ degradation)[30,33] these anions are avoided in this process.

**Table 2:** Asymmetric aza-Diels-Alder reaction of Danishefsky's diene **1** with imine **2** (R = Ph) in the presence of chiral IL **4** (R', X = OH, Y = OTf). Conditions^a^: imine **2**:diene **1**:IL **4** = 1:1.5:2; temperature: 30°C; time: 4.30 h.

Entry	IL **4**, R'	Isolated yield **3** (%)	de **3**^b^ (%)

1	C_4_H_9_	68	45
2	C_8_H_17_	65	60
3	C_16_H_33_	30	72

a) Diene added into reaction medium in three phases: 0.5 equiv. at equal intervals b) de determined by chiral HPLC with a margin of error about 1%.

The next step was to further explore the chiral ILs role as a chiral medium in the asymmetric aza-Diels-Alder reaction. For this purpose, a variety of chiral imines **2**, (derived from aromatic, aliphatic or branched chain aliphatic aldehydes) were tested under similar conditions. The reactions of chiral imines **2** (Table 3) with the diene **1**, generally resulted in cycloadduct **3** in good yield and significant diastereomeric excess. All reactions were performed in the presence of 2 equivalents of chiral IL **4** at room temperature for 4.5 hours. The drop in diastereoselectivity was observed in the case of *p*-nitrophenyl (Table 3, entry 4, R = *p*-NO_2_C_6_H_4_,). This is due to hydrogen bond formation between the OH group of the chiral ionic liquid and the NO_2_ function of the substrate. A similar decrease in asymmetric induction was observed by our group in the mechanistically related Baylis-Hillman reaction between *p*-nitrobenzaldehyde and methyl acrylate using the above chiral IL.[25] A slight increase in de (not optimized value) was detected when employing *p*-methoxyphenyl (Table 3, entry 2, R = *p*-MeOC_6_H_4_). ILs are highly recyclable and do not lose any of their properties even when used four consecutive times (Table 3, entry 1). The results obtained are given in Table 3.

**Table 3:** Asymmetric aza-Diels-Alder reaction of diene **1** with different imines **2**. Conditions^a^: imine **2**:diene **1**:IL **4** (X = OH, R' = C_8_H_17_, Y = OTf) = 1:1.5:2; temperature: 30°C; time: 4.30 h

Entry	R	Isolated yield **3** (%)	de **3**^b^ (%)

1	C_6_H_5_	60 (62, 63, 65)^c^	60 (58, 60, 61)
2	*p*-MeOC_6_H_4_	77	66
3	*p*-ClC_6_H_4_	76	61
4	*p*-NO_2_C_6_H_4_	76	32
5	C_6_H_13_	61	54
6	(CH_3_)_2_CHCH_2_	74	53

a) Diene added into reaction medium in three phases: 0.5 equiv. at equal intervals b) de determined by chiral HPLC with a margin of error about 1%. c) Isolated yields obtained by reaction with recycled IL are given in brackets.

**Figure 1:**
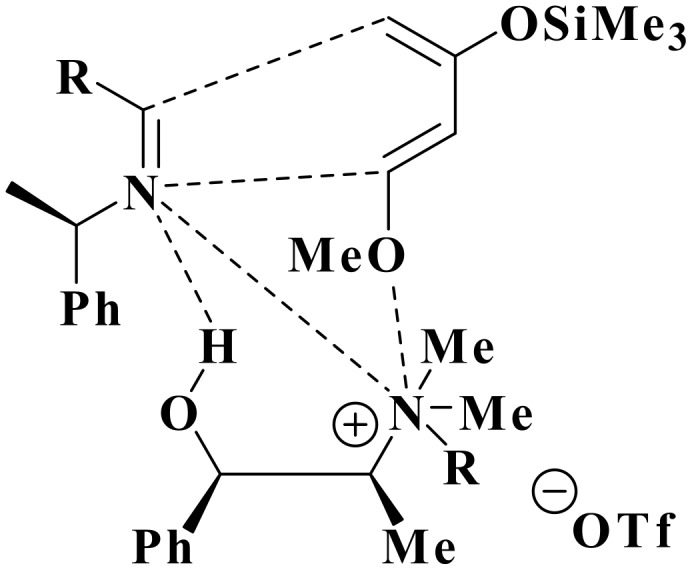
Possible interactions of substrates or intermediate of the aza-Diels-Alder reaction with the chiral cation of an IL containing a hydrogen-bond donor.

Some experiments were carried out to investigate the chirality transfer mechanism by the chiral reaction medium. As mentioned in the literature, the presence of hydroxyl group and the ammonium function are very important for chirality transfer. This had already been reported by Colonna and co-workers[34–35] in the borohydride asymmetric reduction of carbonyl compounds using a chiral phase transfer catalyst. This observation is supported by our studies in the asymmetric Baylis-Hillman reaction.[25] Thus, when (-)-*N*-methylephedrine was used as a chiral source without acid catalyst, no trace of the desired product was observed (Table 4, entry 2), a fact already mentioned in the literature.[36–37] On the other hand, when the hydroxyl group of the chiral IL was replaced by an acetyl group, only a 55% de with 30% yield were detected (Table 4, entry 3). Furthermore, only 32% of de was detected when using chiral imine in the presence of ZnCl_2_ as Lewis acid catalyst (Table 4, entry 4). Lastly, the use of non chiral IL **5**, containing a free hydroxyl function, without acid catalyst, led to the same diastereoselectivity (34%) with 65% in yield, confirming the important effect of the hydroxyl group on reaction yield (Table 4, entry 5). The results showed that not only can chiral ILs be used as solvent and catalyst but also as chiral inductor in the asymmetric aza-Diels-Alder reaction. The key to effective asymmetric induction is strong intermolecular interactions like electrostatic attraction and hydrogen bonding between ionic solvents and intermediates or transition states of the diastereoselective reaction step (Figure 1). This observation was made by our group[25] and further confirmed by Leitner and co-workers.[29] The main results are summarized in Table 4.

**Table 4:** Asymmetric aza-Diels-Alder reaction of diene **1** with imines **2** (R = Ph). Conditions^a^: imine **2**:diene **1**:IL **4** (R' = C_8_H_17_, Y = OTf) = 1:1.5:2; temperature: 30°C; time: 4.30 h

	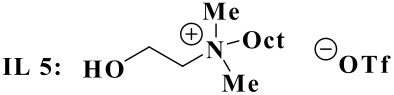		
Entry	Chiral source **4**	Isolated yield **3** (%)	de **3**^b^ (%)

1	**4**, X = OH	66	60
2	(-)-NME^c^	0	0
3	**4**, X = OAc	30	55
4	No **4**^d^	60	32
5	IL **5**	65	34

a) Diene added into reaction medium in three phases: 0.5 equiv. at equal intervals b) de determined by chiral HPLC with a margin of error about 1%. c) (-)-*N*-methylephedrine (1 equiv.) is used as a chiral source. d) ZnCl_2_(10% mol) added.

In conclusion, we are pleased to be able to demonstrate a chemically efficient and cost effective procedure for the diastereoselective synthesis of 2-substituted-2,3-dihydro-4-pyridones derivatives through the asymmetric aza-Diels-Alder reaction under green chemistry conditions. The reaction of Danishefsky's diene with chiral imines has proved to perform better in chiral ionic liquids at room temperature. The entire experiment makes no use of either acid catalyst or organic solvent. At this point in our research, we highly recommend the use of ionic liquids, due to their unique properties and especially their high degree of organization as chiral reaction medium in this reaction. Further investigations to provide useful insights into the understanding of the use of chiral ILs in asymmetric induction are in progress in our laboratory. The results of these studies will be communicated in due course.

## Supporting Information

File 1 Experimental procedures and full spectroscopic data for all new compounds.
