# Supplementary material for: Asymmetric aza-Diels-Alder reaction of Danishefsky's diene with imines in a chiral reaction medium
Source: Beilstein J Org Chem. 2006 Sep 18;2:18. doi: 10.1186/1860-5397-2-18 (PMC1592496; doi:10.1186/1860-5397-2-18)
Supplement: File 1 — Experimental procedures and full spectroscopic data for all new compounds. [file Beilstein_J_Org_Chem-02-18-s001.doc]

**Supporting Information for**

Asymmetric Aza-Diels-Alder Reaction of Danishefsky’s Diene with Imines in a Chiral Reaction Medium

Bruce Pégot, Olivier Nguyen Van Buu, Didier Gori, Giang Vo-Thanh*

Laboratoire de Chimie des Procédés et Substances Naturelles, ICMMO, CNRS UMR 8182, Université Paris-Sud, 91405 Orsay Cedex, France.

*E-mail :* [*gvothanh@icmo.u-psud.fr*](mailto:gvothanh@icmo.u-psud.fr)

**General Considerations**

Melting points were measured on a Kofler bank. The NMR spectra were recorded in CDCl_3_. ^1^H NMR spectra were recorded at 360 or 250 MHz. The chemical shifts (δ) are reported in ppm relative to the TMS as internal standard. *J* values are given in Hz. ^13^C NMR spectra were recorded at 90 or 62.5 MHz. IR spectra were recorded on a FT-IR Perkin-Elmer instrument. TLC was carried out with 0.2 mm thick silica gel plates (GF_254_). Visualization was accomplished by UV light or phosphomolybdic acid solution. The columns were hand packed with silica gel 60 (200-300). Conversions and yields were estimated by GC using octadecyl acrylate as an internal standard. The GC device (GC 9000 series Fisons,) was fitted with a non polar capillary column, film thickness: 0.1 µm, carrier gas: helium. GC equipment fitted with a hardware (NCI 900 series interface) and software (Turbochrom) system developed by Perkin Elmer Co. Diastreomeric excesses were determined by chiral HPLC (Perkin Elmer), which was fitted with a chiral *(S,S*)-whelk-01 column 4.6 mm x 250 mm (thermostatted column 0-1°C).

All reagents and solvents were purchased from commercial sources (Acros, Aldrich) and were used without further purification.

**General Procedure for the asymmetric aza Diels-Alder Reaction of Danishefsky’s Diene with Imines**

A mixture of imine **2** (1 mmol), chiral ionic liquid **4** (2 equiv.) and Danishefsky’s diene **1** (1.5 equiv. added in three phases) was stirred at 30°C for 4.30 hours. The reaction mixture was extracted from the ionic liquid phase with Et_2_O (10 mL x 3). The ether layer was dried over anhydrous MgSO_4_ and evaporated under reduced pressure. The residue was purified by flash column chromatography (AcOEt / pentane = 10 / 90 to 70 / 30) to provide **3**.

The chiral ionic liquid was dissolved in dichloromethane (20 mL) and then recycled by washing with water (10 mL x 2). The organic phase was dried over anhydrous MgSO_4_, filtered and evaporated in *vacuo* to afford the recycled ionic liquid. Spectra data (IR, ^1^H and ^13^C) were identical to the initial ionic liquid sample. This IL was reused without loss of efficiency (Table 3, entry 1).

**Spectral Data for Products**

**(2*S*)-2,3-dihydro-2-phenyl-1-((*R*)-1-phenylethyl)pyridine-4-(1*H*)-one 3a**

Mp: 74°C; [α]_D_^26^: + 183.7 (c 1.96, CHCl_3_) (ed = 97% determined by chral HPLC). IR (neat): 3029, 2975, 1639, 1590, 1494, 1451, 1393, 1294, 1152, 762, 700 cm^-1^. ^1^H NMR (CDCl_3_) δ 1.45 (d, 3H, *J* = 6.8 Hz), 2.55-2.88 (m, 2H), 4.43 (q, 1H, *J* = 6.8 Hz), 4.70 (dd, 1H, *J* = 6.8, 8.8 Hz), 5.04 (d, 1H, *J* = 7.0 Hz), 7.06 (d, 1H, *J* = 7.0 Hz), 7.09-7.42 (m, 10H). ^13^C NMR (CDCl_3_) δ 17.4, 43.2, 59.0, 60.2, 98.0, 125.8, 126.4, 127.3, 128.0, 128.3, 128.8, 138.7, 139.6, 149.0, 189.8. HRMS (EI) calcd. for C_19_H_19_NO (M^+^) 277.1461, found 277.1460.

**(2*R*)-2,3-dihydro-2-phenyl-1-((*R*)-1-phenylethyl)pyridine-4-(1*H*)-one (3a diastereomer)**

IR (neat): 3029, 2975, 1639, 1590, 1494, 1451, 1393, 1294, 1152, 762, 700 cm^-1^. ^1^H NMR (CDCl_3_) δ 1.60 (d, 3H, *J* = 7.0 Hz), 2.55-2.88 (m, 2H), 4.28 (q, 1H, *J* = 6.8 Hz), 4.70 (dd, 1H, *J* = 6.8, 8.8 Hz), 5.14 (d, 1H, *J* = 7.5 Hz), 7.61 (d, 1H, *J* = 7.7 Hz), 7.09-7.42 (m, 10H). ^13^C NMR (CDCl_3_) δ 21.4, 43.9, 59.9, 60.6, 99.5, 125.9, 126.6, 127.8, 128.3, 12873, 128.9, 139.3, 141.8, 152.0, 190.4. HRMS (EI) calcd. for C_19_H_19_NO (M^+^) 277.1461, found 277.1460.

**(2*S,*2*R*)-2,3-dihydro-2-(4-methoxyphenyl)-1-((*R*)-1-phenylethyl)pyridine-4-(1*H*)-one 3b** (two diastereomers are observed)

IR (neat): 3030, 2969, 1639, 1588, 1575, 1511, 1453, 1295, 1250, 1150, 1030, 831, 772, 700 cm^-1^. ^1^H NMR (CDCl_3_) δ 1.48 (d, 3H, *J* = 7.2 Hz), 1.57 (d, 3H, *J* = 7,2 Hz), 2.57-2.83 (m, 2H), 3.83 (s, 3H), 4.31 (q, 1H, *J* = 7.2 Hz), 4.43 (q, 1H, *J* =7.2 Hz), 4.62- 4.67 (m, 1H,), 5.05 (d, 1H, *J* = 7.4 Hz), 5.15 (d, 1H, *J* = 7.4 Hz) 6.87-6.93 (m, 2H), 7.06 (d, 1H, *J* = 7.5 Hz), 7.14-7.17 (m, 2H), 7.27-7.42 (m, 2H), 7.61 (d, 1H, *J* = 7.7 Hz). ^13^C NMR (CDCl_3_) δ 17.4, 21.6, 43.8, 44.1, 55.3, 58.9, 59.7, 60.2, 60.7, 98.2, 99.7, 114.2, 125.8, 127.3, 127.5, 127.9, 128.1, 128.3, 128.7, 128.9, 131.1, 131.3, 139.6, 141.9, 149.3, 152.8, 159.6, 190.1, 190.8. HRMS (EI) calcd. for C_20_H_21_NO_2_ (MH^+^) 308.1645, found 308.1644.

**(2*S,*2*R*)-2,3-dihydro-2-(4-chlorophenyl)-1-((*R*)-1-phenylethyl)pyridine-4-(1*H*)-one 3c** (two diastereomers are observed)

IR (neat): 3030, 2969, 1634, 1569, 1556, 1490, 1454, 1297, 1243, 1149, 1091, 831, 766, 700 cm^-1^. ^1^H NMR (CDCl_3_) δ 1.48 (d, 3H, *J* = 7.2 Hz), 1.57 (d, 3H, *J* = 7,2 Hz), 2.60-2.72 (m, 2H), 4.29 (q, 1H, *J* = 7.0 Hz), 4.41- 4.52 (m, 2H), 4.70 (d, 1H, *J* = 6.8, 8.3 Hz), 5.20 (d, 1H, *J* = 7.5 Hz), 5.33 (d, 1H, *J* = 7.7 Hz), 7.12-7.38 (m, 9H), 7.69 (d, 1H, *J* = 7.5 Hz). ^13^C NMR (CDCl_3_) δ 18.8, 21.5, 42.4, 42.9, 59.5, 60.3, 60.6, 60.7, 98.0, 99.0, 125.9, 127.4, 128.2, 128.3, 128.5, 129.0, 129.3, 134.2, 136.8, 137.5, 139.0, 141.2, 151.0, 153.9, 190.3, 190.6. HRMS (EI) calcd. for C_19_H_18_ClNO (MH^+^) 312.1150, found 312.1154.

**(2*S,*2*R*)-2,3-dihydro-2-(4-nitrophenyl)-1-((*R*)-1-phenylethyl)pyridine-4-(1*H*)-one 3d** (two diastereomers are observed)

IR (neat): 3030, 2927, 1635, 1574, 1520, 1453, 1347, 1288, 1150, 856, 755, 701 cm^-1^. ^1^H NMR (CDCl_3_) δ 1.41 (d, 3H, *J* = 7.0 Hz), 1.56 (d, 3H, *J* = 6.8 Hz), 2.42 (dd, 2H, *J* = 4.25, 16.5Hz), 2.80-2.92 (m, 2H), 4.21 (q, 1H, *J* = 7.0 Hz), 4.40- 4.52 (m, 2H), 4.73 (dd, 1H, *J* = 7.0, 8.3 Hz), 4.96 (d, 1H, *J* = 7.5 Hz), 5.07 (d, 1H, *J* = 8.3 Hz), 7.08-7.46 (m, 9H), 7.61 (d, 1H, *J* = 8.3 Hz), 8.07 (d, 2H, *J* = 8.0 Hz). ^13^C NMR (CDCl_3_) δ 17.9, 21.0, 42.2, 42.6, 58.2, 59.6, 60.8, 61.2, 98.3, 99.0, 123.8, 125.6, 126.4, 126.7, 127.2, 127.3, 128.1, 128.3, 128.6, 128.8, 139.0, 140.9, 146.0, 146.8, 147.1, 148.7, 151.7, 188.3, 185.5. HRMS (EI) calcd. for C_19_H_18_N_2_O_3_ (M^+^) 322.1312, found 322.1314.

**(2*S,*2*R*)-2,3-dihydro-2-hexyl-1-((*R*)-1-phenylethyl)pyridine-4-(1*H*)-one 3e** (two diastereomers are observed)

IR (neat): 3030, 2927, 1634, 1575, 1454, 1395, 1287, 1238, 1200, 763, 700 cm^-1^. ^1^H NMR (CDCl_3_) δ 0.75-0.88 (m, 3H), 1.00-1.26 (m, 6H), 1.49 (d, 3H, *J* = 7.0 Hz), 1.56 (d, 3H, *J* = 7.0 Hz), 1.35-1.60 (m, 2H), 1.75-1.95 (m, 2H), 2.10 (d, 1H, *J* = 16.0 Hz), 2.25 (d, 1H, *J* = 16.5 Hz), 2.49-2.62 (m, 1H), 3.21-3.26 (m, 1H), 3.43-3.47 (m, 1H), 4.30- 4.50 (m, 1H), 4.76 (d, 1H, *J* = 7.5 Hz), 4.90 (d, 1H, *J* = 7.0 Hz), 6.82 (d, 1H, *J* = 7.5 Hz), 7.17-7.34 (m, 5H). ^13^C NMR (CDCl_3_) δ 18.7, 20.5, 21.5, 24.4, 24.5, 27.6, 28.1, 28.4, 28.7, 30.3, 30.6, 37.8, 37.9, 55.1, 60.3, 60.5, 95.4, 95.9, 124.8, 126.2, 127.0, 127.3, 127.6, 127.9, 128.5, 138.9, 141.6, 146.8, 149.3, 189.6, 189.7. HRMS (EI) calcd. for C_19_H_27_NO (MH^+^) 286.2165, found 286.2168.

**(2*S,*2*R*)-2,3-dihydro-2-isobutyl-1-((*R*)-1-phenylethyl)pyridine-4-(1*H*)-one 3f** (two diastereomers are observed)

IR (neat): 3030, 2955, 1634, 1575, 1454, 1395, 1293, 1273, 1217, 766, 700 cm^-1^. ^1^H NMR (CDCl_3_) δ 0.66-0.81 (m, 6H), 0.95-1.15 (m, 1H), 1.48 (d, 3H, *J* = 7.0 Hz), 1.56 (d, 3H, *J* = 7.0 Hz), 1.60-1.68 (m, 2H), 1.83-1.95 (m, 2H), 2.17 (d, 1H, *J* = 16.3 Hz), 2.28 (d, 1H, *J* = 16.5 Hz), 2.48-2.65 (m, 1H), 3.24-3.32 (m, 1H), 3.50-3.56 (m, 1H), 4.35 (q, 1H, *J* = 7.0 Hz), 4.48 (q, 1H, *J* = 7.0 Hz), 4.85 (d, 1H, *J* = 6.7Hz), 5.00 (d, 1H, *J* = 6.7 Hz), 6.88 (d, 1H, *J* = 7.0 Hz), 7.17-7.34 (m, 5H). ^13^C NMR (CDCl_3_) δ 19.3, 20.6, 21.0, 23.0, 23.7, 36.4, 36.9, 38.3, 53.5, 54.2, 60.7, 61.2, 95.7, 96.1, 125.4, 126.7, 127.5, 127.7, 128.4, 128.5, 139.4, 142.1, 147.3, 149.8, 189.8, 190.0. HRMS (EI) calcd. for C_17_H_23_NO (MH^+^) 258.1852, found 258.1861.
